# Applications of Photonic Crystal Nanobeam Cavities for Sensing

**DOI:** 10.3390/mi9110541

**Published:** 2018-10-23

**Authors:** Qifeng Qiao, Ji Xia, Chengkuo Lee, Guangya Zhou

**Affiliations:** 1 Department of Mechanical Engineering, National University of Singapore, Singapore 117579, Singapore; e0204977@u.nus.edu (Q.Q.); e0267876@u.nus.edu (J.X.); 2 Department of Electrical and Computer Engineering, National University of Singapore, Singapore 117583, Singapore; elelc@nus.edu.sg; 3 Center for Intelligent Sensors and MEMS (CISM), National University of Singapore, Singapore 117608, Singapore

**Keywords:** photonic crystal cavity, photonic crystal nanobeam cavity, optical sensor, refractive index sensor, nanoparticle sensor, optomechanical sensor, temperature sensor

## Abstract

In recent years, there has been growing interest in optical sensors based on microcavities due to their advantages of size reduction and enhanced sensing capability. In this paper, we aim to give a comprehensive review of the field of photonic crystal nanobeam cavity-based sensors. The sensing principles and development of applications, such as refractive index sensing, nanoparticle sensing, optomechanical sensing, and temperature sensing, are summarized and highlighted. From the studies reported, it is demonstrated that photonic crystal nanobeam cavities, which provide excellent light confinement capability, ultra-small size, flexible on-chip design, and easy integration, offer promising platforms for a range of sensing applications.

## 1. Introduction

Currently, optical sensors are among the most widely used types of sensing platforms for various applications in every aspect of life, including industry, society, and the military. Optical sensors have advantages over other types of sensors including small size, usability in harsh environments, remote sensing, immunity to interference, etc. With the recent advance of studies on optical microcavities [1], optical sensors can also be realized through on-chip microcavities. Through resonant recirculation, light can be confined into a small volume by optical microcavities, among which photonic crystal cavity is a promising candidate for sensing applications due to its small mode volume and strong light field confinement [2]. They have received much attention in recent years due to the flexible structure, easy on-chip integration, outstanding light confinement capability, and compact size [3]. 

In this review, we focus on the sensing applications of photonic crystal nanobeam cavities [4,5,6,7,8,9,10,11,12,13,14,15,16,17,18,19,20,21,22,23,24,25,26,27], which are attractive for their ultra-small physical footprint and extremely low effective mass. This review is organized as follows. Section 2 gives a brief overview of photonic crystals and other types of optical cavities. Section 3 outlines the sensing principle and highlights some key developments in refractive index sensing based on nanobeam cavities. Section 4 presents applications on nanoparticle sensing and analyzes the techniques for nanoparticle capture. Section 5 outlines the mechanisms for optomechanical sensing and at the same time introduce their applications. Section 6 highlights the principles and applications of temperature sensing using photonic crystal nanobeam cavities. Finally, we sum up the whole review. 

## 2. Optical Cavity

Optical microcavities are capable of confining light in small mode volumes. Based on them, sensors are possible to achieve unprecedentedly high sensitivity and low detection limits. The resonance characteristics of a microcavity, such as resonance wavelength and line width, can be significantly affected by slight physical and chemical variations in the optical mode region. 

**Figure 1:**
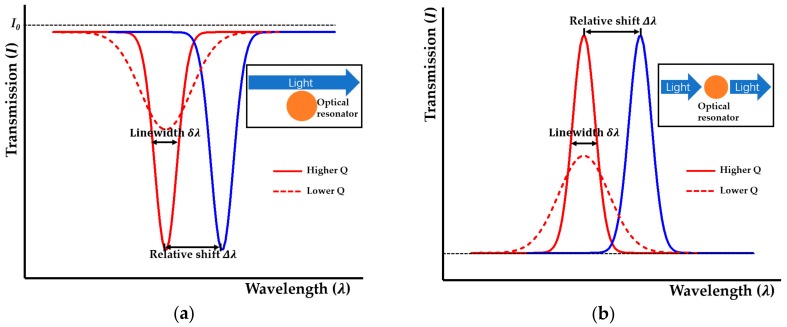
Transmission spectrum of an optical microcavity: (**a**) a characteristic dip at resonance wavelength for side-coupled cavity; (**b**) a characteristic peak at resonance wavelength for the input-cavity-output configuration. The resonance wavelength shifts from the red line to blue line due to the physical or chemical variations in optical mode region. In addition, the decrease of *Q* factor causes the expansion of spectral resonance line width (*δλ*).

In a photonic cavity, only light at its resonance wavelengths can be strongly coupled into it. For a whispering gallery mode (WGM) cavity or a Fabry-Pérot (F-P) cavity, the resonance wavelength *λ_r_* is defined by:

(1) $$\lambda_{r}=Ln_{eff}/m$$

where *n_eff_* is the effective refractive index of the cavity, *L* is the round-trip optical path length and *m* is an integer. A resonance wavelength shift can be induced if there is a change of effective refractive index in the optical mode region. As light propagates through the cavity, a dramatic dip or peak in the intensity of the transmitted light occurs, which can be monitored in the transmission spectrum. For general sensing applications, measurements on the shift of resonance wavelength (*Δλ*) are most frequently implemented (Figure 1). The transmission spectrum of a side-coupled optical resonator is shown in Figure 1a. In Figure 1b, the transmission spectrum of an optical resonator that is directly in the optical path (i.e., input-cavity-output configuration) is presented. In addition to resonance wavelength, the quality factor (*Q*) is used to compare losses in optical microcavities, which is defined as:

(2) $$Q=\lambda_{r}/\delta \lambda$$

where *δλ* is the resonance linewidth. The *Q* factor indicates the photon lifetime within the cavity. *Q* factors can range from 10^3^ to 10^10^ [28] for different cavity designs. For high-*Q* microcavities, continuous and repetitive sampling of analytes at the resonator surface contributes to the high sensitivity of optical microcavities by significantly increasing the effective optical path length for light matter interaction. 

Following extensive studies on optical cavity designs and technological advance in device fabrication, a variety of micro- and nano-photonic cavity structures have been developed. Below, some of the typical ones are introduced.

**Figure 2:**
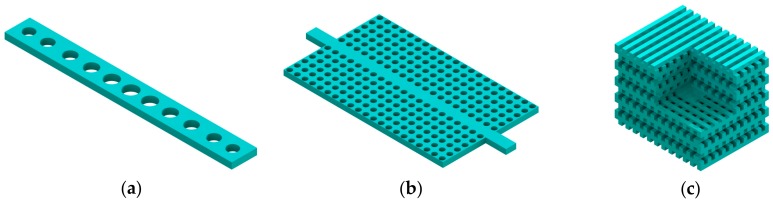
Schematic diagrams of cavities using (**a**) 1D, (**b**) 2D, and (**c**) 3D photonic crystals (PhCs).

Photonic crystal (PhC) microcavities possess regions of varying materials with different refractive indices arranged in a periodic structure and exhibit abundant optical properties of slowing down and confining the light. The periodic spatial arrangements of contrasting dielectric media create a photonic bandgap (PBG) [29,30]. Through periodic modulation of the dielectric constant in one, two, or three orthogonal directions in a structure, PhC can be obtained. 1D PhCs can be formed by placing alternating dielectric stacks periodically [31] or by etching a row of holes in a perfect waveguide (in Figure 2a). 2D PhCs can be realized either by growing high aspect ratio dielectric rods or by etching holes in a higher dielectric material periodically in two dimensions [32]. The latter is most commonly adopted due to its easy fabrication process. In 3D PhCs, a complete PBG can be obtained and the refractive index is modulated in all three directions [33,34]. The fabrications for this type of structures are challenging. The micro-/nano-photonic cavities constructed using these three types of PhCs are schematically shown in Figure 2. 

In 1997, Foresi et al. [35] demonstrated the proof of concept of a nanobeam cavity through integrating PBG structures directly into a silicon waveguide on a silicon-on-insulator (SOI) wafer. This nanobeam cavity had a modal volume (*V*) of 0.055 µm^3^ and a *Q* factor of 265 at resonance wavelength 1.56 µm. The high *Q*/*V* ratio and large bandgap of this proposed PBG waveguide microcavity made it advantageous over traditional stacked mirror cavities. In general, the *Q* factor and modal volume are used to characterize optical cavities, and high *Q*/*V* is desirable for many applications such as filter, laser, and high Purcell factor [36]. Later, much work has been carried out experimentally and numerically in order to increase the *Q*/*V* ratio through subtle tuning of the hole geometry around the cavity defect [37,38,39,40,41,42,43]. Importantly, Lalanne et al. proposed the Bloch mode engineering concepts [42] and revealed two physical mechanisms of the fine-tuning of holes geometry of PhC cavities [43]. The first mechanism could be realized through engineering the mirrors and thus reducing the out-of-plane far field radiation. The other mechanism involved recycling, which could be understood as an interference between leaky modes and fundamental modes. Moreover, the authors modified a classical F-P cavity model with consideration of energy recycling through leaky waves to physically interpret the second mechanism. As shown through the analytical model, the recycling mechanism complied with a phase-matching condition. Through subtle tapering of the hole size, the modal mismatch effects between cavity defect space and PhC mirrors could be reduced, which could increase the *Q*/*V* ratio by several orders of magnitude. These studies provide significant physical insights on the optimization of nanobeam cavity and are fundamental for further development of nanobeam cavity design. 

**Figure 3:**
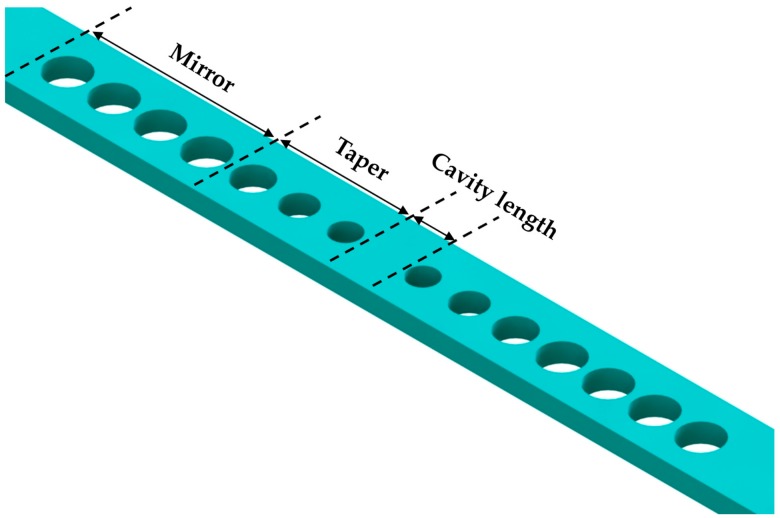
Three elements involved in the optimization of PhC nanobeam cavities.

Based on the Bloch mode engineering concepts [42,44], in 2006 Velha et al. [45] achieved a nanobeam cavity with *Q* factor of 8900. Furthermore, in 2007 Velha et al. [46] tried to adjust the length of the cavity defect as a function of the number of mirror holes. Consequently, they obtained a modal volume of 0.6 (λ/n)^3^ and a *Q* factor of 58,000. In addition, many studies have been carried out on the design concepts and methodologies for the optimization of nanobeam cavities [47,48,49,50,51,52,53,54]. In general, three elements are modulated in the design process, the PhC mirror, cavity length, and taper (in Figure 3). Notomi et al. [48] proposed mode-gap-based cavities numerically in 2008, and later Kuramochi et al. [51] experimentally demonstrated the ladder nanobeam cavity and stack nanobeam cavity on an SOI wafer with a *Q* factor of higher than 10^5^ and a small modal volume. Later, a deterministic method was proposed for a nanobeam cavity with high *Q*/*V* [52,53]. The authors adopted photonic band calculations rather than a trial-based method in the design process, which could save on computation costs and improve the design efficiency. In this way, the final cavity resonance with a small deviation from the predetermined wavelength could be achieved. 

**Figure 4:**
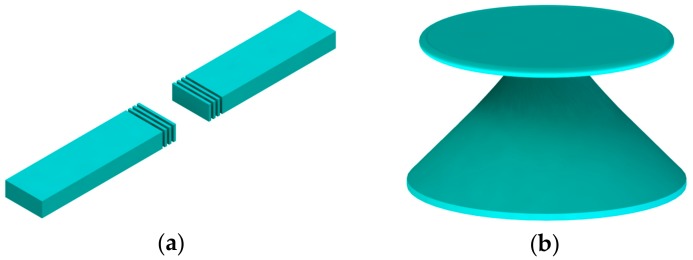
(**a**) Schematic diagram of Fabry-Pérot (F-P) cavity with DBRs; (**b**) schematic diagram of whispering gallery mode (WGM) cavity.

In addition, there are some other types of optical microcavities using different confinement methods. As shown in Figure 4a, two planar mirrors are placed parallelly to form an F-P cavity. In this way, resonant photons can be bounded between mirrors so that light can be confined to the optical cavity. Due to the unique “air gap” offered by the F-P cavity, it has some advantages over other optical cavities, such as tunable cavity length and easy interaction with analytes. In WGM microcavities (Figure 4b), the circular structure of dielectric material can confine optical field strongly through total internal reflection [55]. WGM cavities with an ultra-high optical *Q* (exceeding 10^8^) [28] provide a remarkable advantage over other microcavities in terms of extremely low loss and long photon lifetime.

## 3. Refractive Index Sensing

Refractive index (RI) sensing is one of the most prominent commercialized sensing technologies, and there is a vast amount of literature on RI sensors. Various examples of RI sensors using photonic structures such as ring resonators [56,57], long-period fiber gratings [58,59], surface plasmon resonators [60], and PhCs have been proposed in recent years. In this section, we focus on RI sensors using PhC nanobeam cavities. 

### 3.1. Sensing Principle

For sensing applications, the resonance wavelength shift of a PhC cavity induced by RI changes in the optical mode region can be measured to evaluate the RI variations. Section 3 gives an overview of RI sensors for detection of the homogenous change of background RI in the optical mode region, which is usually used for the determination of RI changes in liquid or gas samples.

With the use of perturbation theory, the frequency shift *Δω* caused by a small perturbation of dielectric function *Δε* can be obtained as follows [32]:

(3) $$\Delta \omega =-\frac{\omega }{2}\frac{\int d^{3}r\Delta \varepsilon (r){\left|E(r)\right|}^{2}}{\int d^{3}r\varepsilon (r){\left|E(r)\right|}^{2}}+O(\Delta \varepsilon^{2}),$$

where *E*(**r**) is the mode profile of the perfectly linear and unperturbed dielectric function *ε*(**r**). Writing Δε≈ε⋅2Δn/n and considering the homogenous change of RI, which means that *Δn/n* is all the same in the perturbed region and zero in the unperturbed region. An intuitive interpretation of Equation (3) is then given as [32]:

(4) $$\frac{\Delta \omega }{\omega }\approx -\frac{\Delta n}{n}\overset{}{}(fraction\;of_{}\int \varepsilon {\left|E\right|}^{2}\;in\;the\;perturbed\;regions).$$

It is indicated in Equation (4) that the frequency change is proportional to the fraction of electric field energy confined in the perturbed region.

Furthermore, for detection of a nanoparticle or a single molecule in the vicinity of the photonic cavity, the cavity resonance wavelength shift can be given as follows [15]:

(5) $$\frac{\delta \lambda }{\lambda }=\frac{3(\varepsilon_{p}-\varepsilon_{s})}{\varepsilon_{p}+2\varepsilon_{s}}\frac{{\left|E_{mol}\right|}^{2}}{2\int \varepsilon {\left|E\right|}^{2}dr}V_{mol},$$

where *δλ* is the shift of resonance wavelength, *ε_s_* is the permittivity of the background environment, *ε_p_* is the permittivity of the nanoparticle, **E***_mol_* is the electric field at the nanoparticle location, $\int \varepsilon {\left|E\right|}^{2}dr$ is the overall optical mode energy inside cavity, and *V_mol_* is the volume of the nanoparticle. From Equations (4) and (5), it is indicated clearly that a cavity with a small mode volume and a strong optical field concentrated at the perturbed (or sensing) region is preferred to yield a large resonance shift for high sensitivity.

To quantitatively compare and evaluate the capability of the RI sensor, the concepts of sensitivity and detection limit are introduced [61]. The sensitivity is defined as the resonance shift with unit variation of sample RI, and the detection limit is defined as the minimal variation of RI that can be measured precisely. Various RI sensors based on PhC nanobeam cavities have been put forward to achieve outstanding refractive sensing capability in the past decade. The developments of these designs will be reviewed in detail.

### 3.2. Sensing Applications

Initial work on PhC RI sensors adopted primarily 2D PhC cavities [62,63]. Chow et al. [62] experimentally demonstrated the measurement of the shift in resonant wavelength of a 2D PhC microcavity induced by the change of ambient RI. Commercially available optical fluids having different refractive indices were used for experimental characterization. The proposed sensor with a *Q* factor of 400 demonstrated a sensitivity of 200 nm/RIU and a detection limit of 0.002 RIU. A growing number of studies on RI sensors have focused on 1D PhC cavities in the last decade due to the fact that PhC nanobeam cavities have demonstrated ultra-high *Q* factors experimentally [48]. What is more, PhC nanobeam cavities show clear advantages in miniaturization and on-chip integration of optical sensors due to their small effective masses and physical footprints.

#### 3.2.1. Efforts on Sensitivity

As introduced in Section 2, most of the initial work on nanobeam cavity design has been motivated by the development of optical communications and data processing. Thus, many studies have focused on the improvement of *Q*/*V* ratio, which is crucial for functional devices such as optical filter and optical switch. Meanwhile, some efforts have been carried out to use these nanobeam cavities as sensors. For the use of sensing, the design objective is not limited to high *Q*/*V* ratio, and many studies have been put forward to optimize the cavity design for the sensing performance.

**Figure 5:**
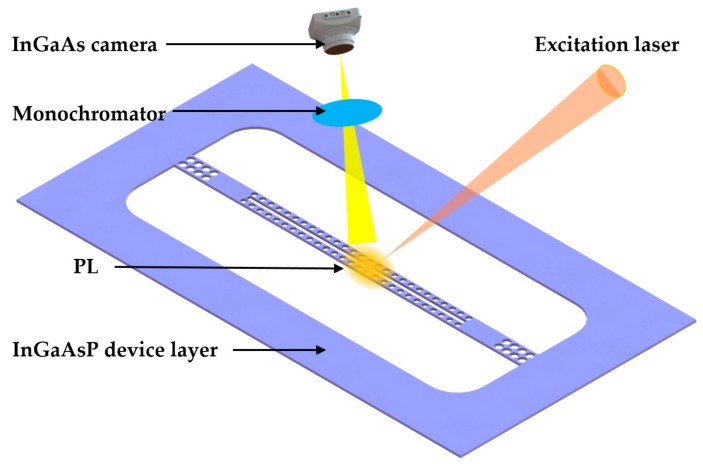
Refractive index (RI) sensor based on PhC slot nanobeam slow light waveguide.

Wang et al. [5] demonstrated the experiment of RI sensing with the use of a single nanobeam cavity in 2010. As shown in Figure 5, the PhC nanobeam waveguides were designed to be two parallelly suspended nanobeams with a small slot between them. Moreover, these two nanobeams were patterned with 1D holes for strong light confinement in the slot region. Significantly, the light field is confined in the low RI region. Due to a large overlap with the potential analyte, the fabricated sample could obtain a sensitivity of 700 nm/RIU and a *Q* factor of 500 at 1386.5 nm. The parameters of the PhC structure were determined using a photonic bands software package [64]. Furthermore, the proposed sensor was evaluated theoretically through the use of a 3D finite-difference time-domain (FDTD) method [65] and analyzed experimentally after fabrication, which were useful approaches for the characterization of the proposed sensor. For the experimental demonstration, the proposed sensor was fabricated on an InGaAsP membrane of 220 nm thickness with embedded InAs quantum dots (QD). This could easily yield photoluminescence (PL) after excitation with a continuous wave (CW) diode laser [66]. After being dispersed through a monochromator, the PL signal was detected with a liquid-nitrogen-cooled InGaAs camera. 

With the development of nanobeam cavity design methods, there have been some ultra-high *Q* nanobeam cavities emerging. Typically, high-*Q* cavities are preferred for RI sensing due to the advantageous detection limit. However, due to the lack of light-matter interaction, an ultra-high *Q* factor nanobeam cavity [67] provided a sensitivity of 83 nm/RIU. This also pointed out the importance of sufficient overlap between optical mode field and analytes (typically having a low index) for sensitivity. 

**Figure 6:**
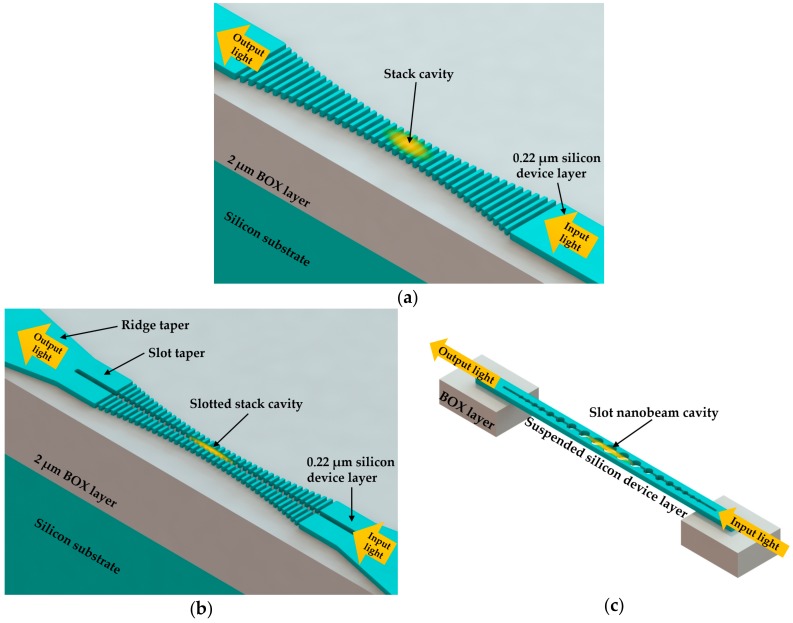
RI sensors aimed at increasing light matter interaction: (**a**) width-modulated stack nanobeam cavity; (**b**) slotted width-modulated stack nanobeam cavity; (**c**) slotted tapered-hole nanobeam cavity.

In order to achieve high sensitivity, Yao and Shi [6] designed a 1D PhC stack mode-gap cavity with width modulation, which localized 35% of the electric field in the low index region. Consequently, the measurement of sensitivity was reported as 269 nm/RIU. The proposed sensor had a wide sensing range. After being immersed in a water-ethanol mixture in their experiment, the proposed sensor still maintained a *Q* factor of about 27,000 across a 50 nm wide spectral band. Furthermore, their structure design was appropriate for the implementations of sensing in a flowing sample. When a PhC nanobeam cavity used for RI sensing, the structures of resonators (high index material) usually isolate the void space (low index region) into pieces, which may potentially block the flow channel of the sample [68]. It can be seen in Figure 6a that the structure of this PhC nanobeam provides enough channels for the flow of the samples.

To further increase sensitivity, some efforts have introduced discontinuity into photonic structures. As the introduced discontinuity results in a high-index-contrast interface in the optical field, the electric field will be much stronger in the low-index region as a large discontinuity to satisfy the continuity of the electric flux density, as stated by the Maxwell Equations [69]. In 2013, Xu et al. [70] made use of the discontinuity based on the modulated width stack cavity design. As shown in Figure 6b, a slot was introduced between periodic arrays of stacks. This design combines the characteristics of a slot waveguide (light field concentrated in the slot region) and a 1D PhC cavity (light field enhanced and confined in the cavity region). The middle slot created a high-index-contrast interface in the optical mode. In this way, the majority of the light field was confined in the slot (low index region) and strongly interacted with analytes. A high sensitivity of 410 nm/RIU could be obtained in the experiments with NaCl solutions of different concentrations. In experiments, the slot tapers and ridge tapers were used to efficiently guide the light into and out of the sensing region. Through the use of numerical simulations, the authors also discovered that the sensitivity increased and the *Q* factor decreased exponentially with the expansion of the slot width. After a tradeoff between *Q* factor and sensitivity was made, the *Q* factor remained at around 10^4^ in their experiments with the cavity in the NaCl solution. Moreover, in 2015 Yang et al. [71] reported a slot PhC nanobeam cavity, as shown in Figure 6c. With the parabolically tapered air holes and an air slot introduced in the middle of the nanobeam, the nanobeam cavity could confine optical field robustly in the low RI region between air holes. Therefore, an ultra-high *Q* factor of 2.67 × 10^7^ and sensitivity of 750.89 nm/RIU could be obtained simultaneously according to their simulation results. What is more, this proposed geometry significantly provided ultra-small mode volume around 0.01 (λ/n_air_)^3^, which made it a potential platform for energy-efficient single particle detection.

#### 3.2.2. The Pursuit of Both High Q Factor and High Sensitivity

As mentioned above, a discontinuity in photonic structures could be adopted to enhance the sensitivity for a dielectric mode (optical field confined in the dielectric waveguide medium) PhC nanobeam. However, this leads to an intrinsic tradeoff between the quality factor and sensitivity for the design of a dielectric mode PhC nanobeam RI sensor [70]. The figure of merit (*FOM*) was proposed by Leif et al. [72] for the evaluation on sensing performance of RI sensors. It can be understood as:

(6) $$FOM=S\cdot Q/\lambda_{res},$$

where *λ_res_* is the resonant wavelength of the cavity, *Q* is the quality factor of the cavity, and *S* is the sensitivity of the cavity. However, for the dielectric mode nanobeam cavity, *FOM* is limited by the tradeoff between the sensitivity (*S*) and quality factor (*Q*). The high sensitivity of RI sensors requires a large overlap between the optical mode field and analytes, which means that the optical mode should be confined in the analyte region (usually a low-index region), while the high *Q* factor of RI sensors requires strong confinement of optical mode in the waveguide medium (usually the high-index region). The high-*Q* cavities are desirable for a low detection limit of RI sensing, because a high *Q* factor leads to narrow resonance linewidth and hence small resonance shift resolution. Therefore, some studies have been carried out aiming at simultaneously achieving ultra-high quality factor and sensitivity [7,73,74]. 

**Figure 7:**
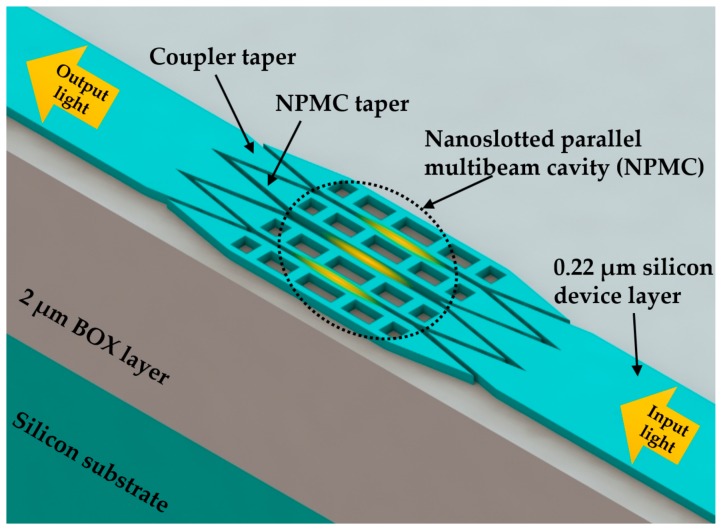
Nanoslotted parallel quadra-beam cavity.

A photonic nanobeam structure with simultaneously ultra-high *Q* and *S* for RI sensing was reported in 2013 by Yang et al. [7]. There were small gaps between these parallel PhC nanobeam cavities. After guided directly into these gaps through coupler tapers, the light was confined in the low-index region. With the use of a deterministic high-*Q* nanobeam cavity design method [53], the resonance could be predicted with the numerical simulation on band-edge frequency of a single unit cell with low computational cost. As the simulation results demonstrated, the nanoslotted parallel multibeam cavity achieved a *Q* factor of 10^7^ and a sensitivity of 800 nm/RIU at telecom wavelength range in liquid with negligible absorption. As a follow-up study, Yang et al. [74] experimentally demonstrated the sensing performance of the proposed sensor in 2014. The device was fabricated on an SOI wafer without release of the buried oxide (BOX) layer, as shown in Figure 7. In the measurement of quadra-beam PhC cavity in ethanol/water solution, the sensor obtained a high-*Q* factor of 7015 and a high sensitivity of 451 nm/RIU experimentally and achieved an *FOM* of 2060. Furthermore, an ultra-low concentration of 10 ag/mL streptavidin could be detected with this proposed sensor in experiments. 

**Figure 8:**
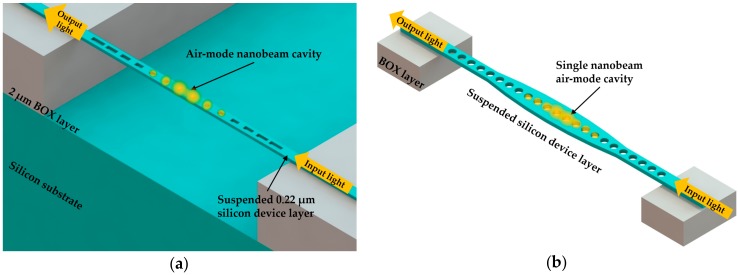
Air mode nanobeam cavities that localize light in low index region: (**a**) nanobeam cavity with modulated air holes; (**b**) nanobeam cavity with modulated width.

Due to the intrinsic tradeoff between *Q* factor and sensitivity of dielectric-mode nanobeam cavity, some researchers extended studies to air mode cavities besides the common focus on dielectric mode cavities. As one of the drawbacks of the multibeam cavities is the relatively large footprint, single nanobeam cavities are expected to have better sensing performance. Quan and Loncar [53] proposed a deterministic design method for a single air mode nanobeam cavity. The air mode cavity could localize optical field in the region of low RI, and thus the air mode cavity with both high *Q* factor and high sensitivity became an appropriate choice for RI sensing. With use of the same design principle for the ultra-high *Q* dielectric-mode nanobeam cavity, the mode at the air band edge could be pulled down into the bandgap to create an ultra-high *Q* air mode nanobeam cavity. As introduced in this study, the dielectric mode nanobeam cavity was achieved through decreasing the size of holes from center to end, while the air mode nanobeam cavity was obtained by increasing the size of holes from center to end. Liang and Quan [8] experimentally demonstrated the air mode PhC nanobeam cavity, which had the advantages of both ultra-high *Q* (2.5 × 10^5^) and ultra-small mode volume (0.01 (λ/n_air_)^3^) at telecom wavelength, in 2015. As shown in Figure 8a, the lengths of these rectangular slots were tapered from the end to the middle, with constant width and periodicity. The proposed sensor was fabricated on an SOI wafer with input and output waveguide. The ultra-small mode volume of this proposed sensor enabled its application to single nanoparticle detection. What is more, the air mode nanobeam cavity can also be realized by tapering the nanobeam width rather than the hole size [53]. Figure 8b presents a RI sensor based on this kind of nanobeam cavity, which was reported by Yang et al. in 2015 [9]. The air mode nanobeam cavity greatly increased the interaction between the optical field and the analytes; thus, the single nanobeam could realize a high sensitivity of 537.8 nm/RIU and a high *Q* factor of 5.16 × 10^6^, as indicated in their simulation results. Based on the similar sensing principle, Huang et al. [75] also reported a tapered width nanobeam cavity with elliptical holes in 2016. Moreover, in 2015 Fegadolli et al. [75] utilized the air-mode nanobeam cavity integrated with a NiCr microheater to demonstrate an RI sensor with a local heating function. The proposed sensor presented a sensitivity of 98 nm/RIU and a heating temperature range of 98 °C, which could be used for sensing applications that required local temperature control [76]. 

In addition to the work described above, some studies have indicated that the optical field in the low-index sensing region could also be enhanced with coupled optical resonators, such as a side-coupled nanobeam cavity, microring resonator, etc. [77,78,79,80]. Furthermore, research has been carried out to enhance the single nanobeam cavity design in order to achieve the ultra-small mode volume suitable for nanoscale RI sensing [81,82,83,84]. Along with much work on telecom wavelength range, Liu et al. [85] experimentally demonstrated a Si_3_N_4_ PhC nanobeam cavity for highly sensitive RI sensing at visible spectrum resonance wavelength. Visible light sensors have the unique advantage of avoiding high light absorption of water in the telecom near-infrared region. In addition, Xu et al. [86] theoretically presented a nanobeam cavity sensor design with a resonant wavelength of 4132 nm in the mid-infrared region for RI sensing. The proposed sensor achieved sensitivity as high as 2280 nm/RIU theoretically.

#### 3.2.3. Multiplex RI Sensing

**Figure 9:**
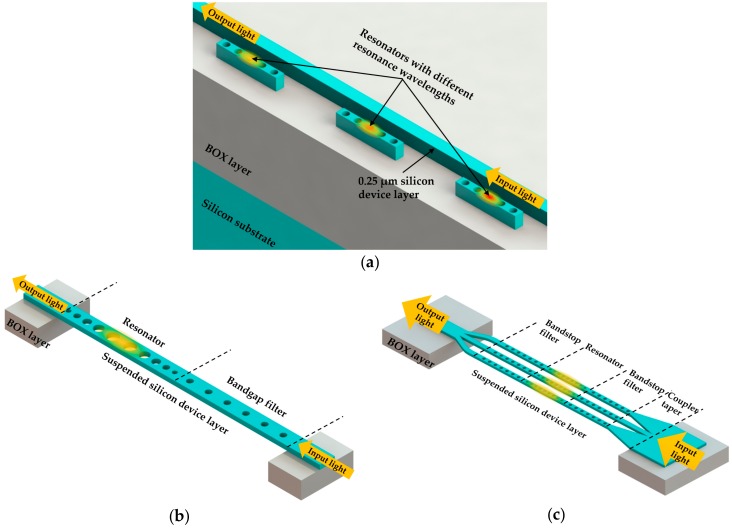
Proposed sensors for multiplex sensing: (**a**) waveguide side-coupled nanobeam cavity array; (**b**) schematic of nanobeam cavity integrated with bandgap filter; (**c**) parallel multiplex sensing array integrated with band stop filters.

Besides much research work on the detection of single analyte, there are studies focused on multiplex RI sensing, including nanobeam cavity sensor array [4] and single nanobeam cavity [11,87]. Mandal and Erickson [4] demonstrated a sensor array based on PhC nanobeam cavities in 2008. A nanobeam cavity with *Q* factor of 8900 was achieved by Velha et al. [88] in 2006 using Bloch-mode engineering concepts. Based on the same design principles, the authors adopted FDTD simulations to optimize the cavity and achieved a *Q* factor of 2000, which was satisfactory for concept illustration in this study. In their design, the nanobeam cavities side-coupled to the input/output waveguide were designed to be at different resonances and separated in different fluid channels. Thus, each analyte could be detected simultaneously with the resonance shift of its corresponding nanobeam cavity by monitoring the output of the waveguide. As shown in Figure 9a, the proposed device was fabricated in a 250 nm thick silicon device layer on an SOI wafer. To demonstrate the nanobeam cavity sensor array, the authors used soft lithography with PDMS to form fluid channels that could separate each nanobeam cavity along the waveguide. The authors injected fluids into the channels and measured the resonance shifts of the corresponding nanobeam cavities in the multi-peak transmission spectrum of the device. Moreover, the effects of functionalized surfaces on low mass detection were also investigated numerically. In addition, some researchers have also demonstrated the improved sensor arrays based on nanobeam cavities side-coupled in series with a waveguide as well as parallel nanobeam cavities directly coupled to the input and output waveguides, as shown in Figure 9c [27,89,90]. As for the multiplex RI sensing with the use of multiple parallel nanobeam cavities, it is possible for the sensing signal of each nanobeam cavity to interfere with others due to the existence of multiple resonances of each nanobeam cavity. Therefore, several studies have been carried out on filtering out the resonances of unwanted orders of a single nanobeam cavity without sacrificing the sensing performance [91,92,93]. The schematic of a typical approach is shown in Figure 9b. With the use of a PhC nanobeam bandgap filter, the free spectral range of each sensing channel could be increased in the wavelength-multiplexed sensing scheme so that crosstalk among multiple channels could be avoided [10], as shown in Figure 9c.

What is more, the single nanobeam cavity can be used to realize complex RI sensing (detection of both real and imaginary parts of RI) [11,87]. In the field of RI sensing based on PhC nanobeam cavity, the majority of studies have focused on the real part of RI, while Zhang et al. [11] in 2016 demonstrated the possibility of multi-element mixture detection based on the combination of both real and imaginary parts of RI detection. In their demonstration of the detection of a D_2_O/H_2_O/EtOH mixture, the authors achieved a sensitivity of 58 nm/RIU for the real part and 139 nm/RIU for the imaginary part, with a satisfactory detection limit. The authors suggested that the changes in real and imaginary parts of RI resulted in linear changes of the resonance wavelength and mode linewidth, respectively. Thus, the proposed sensor was capable of detecting ternary mixture concentrations with two unknown parameters. After the characterization of the sensor responses of resonance wavelength shift and linewidth variation to the known concentrations in specific calibration binary mixtures, the detection of unknown concentrations of a ternary mixture could be realized with the measurements of the total resonance wavelength shift and linewidth change. The dielectric nanobeam cavity with both high transmission and high *Q* factor [94] was fabricated with a silicon device layer of 260 nm thick above the 2 µm thick BOX layer on a SOI wafer, and light was coupled into the cavity through a grating coupler and tapered waveguide. 

### 3.3. Discussion

Many efforts have been carried out to optimize the nanobeam cavity design for RI sensing. For RI sensing applications, there are design objectives other than the high *Q*/*V* ratio. In order to simultaneously achieve outstanding sensitivity and detection limit, studies have worked to increase the light-matter interaction without sacrificing the *Q* factor in the nanobeam cavity design. Many meaningful approaches have been presented, such as the introduction of discontinuity into PhC structures, coupling of light into mirror gaps, and design of air mode cavity. 

In summary, with the advancements of nanobeam cavity design and fabrication in recent years, the PhC nanobeam cavities have become an excellent platform for RI sensing due to the high *Q* factor, small mode volume, and large overlap between light field and analytes. Moreover, the advantage of the small footprint makes it suitable for multiple channel sensing using a wavelength multiplexing scheme and facilitates further on-chip integration. 

## 4. Nanoparticle Sensing

**Figure 10:**
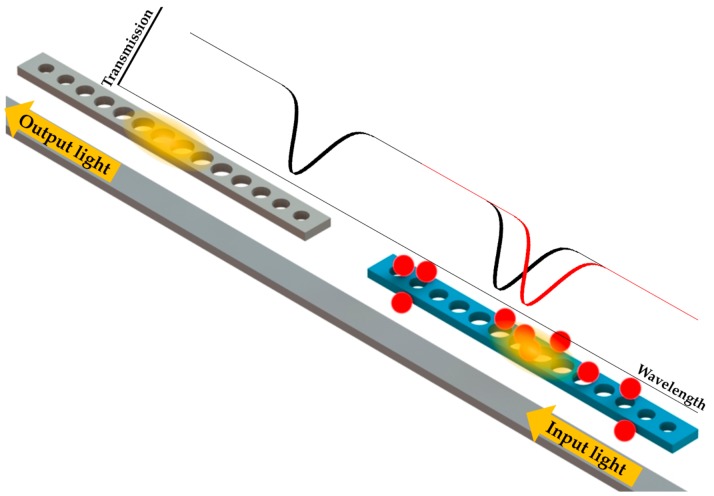
Schematic of nanoparticle sensing.

In the previous section about RI sensors, the studies introduced mainly focus on the homogenous change of RI induced by the sample. Despite some designs already possessing nanoparticle detection ability, we feel that nanoparticle sensing using nanobeam cavities deserves special attention here. For the sensing of nanoparticles such as biomolecules, specific capture of nanoparticles on the sensor/cavity surface is required to induce a significant change in cavity characteristics. As shown in Figure 10, it is hard to induce a detectable resonance shift in a nanobeam cavity without surface binding capability, especially for single nanoparticle sensing.

In recent years, there has been great progress in nanoparticle sensing using micro- and nanoscale optical approaches. To realize next-generation clinical diagnostic tests, much work has been carried out on approaches to sensitive detection of biomolecules, such as DNA, proteins, and other nanoparticles [95]. The detection limit of these nanoparticle sensors is expected to be ultra-low for single molecule capability. Moreover, it is crucial to achieve this detection in an aqueous environment with a selective detection capability among multiple species of nanoparticles. Among all the detection methods reported, the most prominent include those based on resonant cavities [96,97], surface plasmon resonance [98,99], interferometry [100,101], and photonic crystals [102]. Some nanoparticle sensing mechanisms such as mode shift, mode broadening, and mode splitting have been demonstrated using WGM cavities [103,104,105]. PhC nanobeam cavities, which can provide an ultra-small mode volume and high *Q* factor [1], are well suited for light field interaction with ultra-small nanoparticles and biomolecules, and hence show promise in this field.

The sensitivity of the nanoparticle sensor is given as the ratio of the resonance shift induced to the surface density of the captured nanoparticles [61]. This clearly indicates demand for ultra-small mode volume cavity to interact with the target nanoparticles. Moreover, the ability of the sensor surface to capture nanoparticles is important for the sensing performance. This nanoparticle capturing ability can be realized through functionalized coatings on sensing resonators or other techniques such as trapping using optical forces. To realize optimal and selective binding of nanoparticles on the sensor surface, specified functionalized coatings are widely used in many studies. For example, the antibody-antigen locking mechanism is adopted for the binding of target biomolecules to the surface. On the other hand, different techniques such as optical trapping also exist to capture the target nanoparticles. With the use of it, there is no need for coating on the resonator surface; instead we use optical trapping force to achieve the same task. The following will briefly discuss the nanoparticle sensors using these methods. 

### 4.1. Functionalized Coating Surface

**Figure 11:**
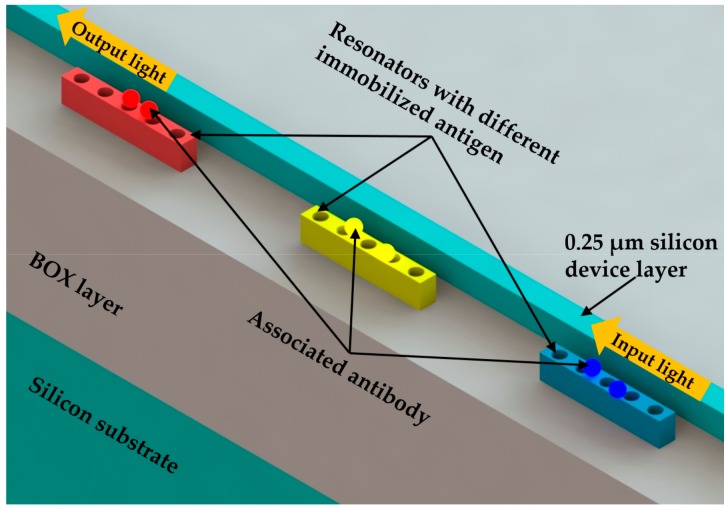
Three waveguide-coupled nanobeam cavities with different immobilized antigens on their surfaces.

The antibody-antigen locking mechanism has been widely adopted for the functionalization of the nanobeam cavity surface. In 2009 Mandal et al. [13] presented a biomolecular sensor with a 1D PhC nanobeam cavity array. The authors demonstrated the capability of this proposed sensor for wavelength-multiplexed sensing with monoclonal antibodies to interleukin 4, 6, 8 on three adjacent nanobeam cavities. As shown in Figure 11, these nanobeam cavities were given different types of functionalized surfaces to capture the corresponding biomolecules. Wavelength-scale mode volume could be obtained due to the optimized PhC structures [88]. The detection limit on the order of 63 ag total bound mass could be achieved according to the estimation using a polyelectrolyte “layer-by-layer” growth model. The proposed device was fabricated in a 250 nm thick silicon device layer on a SOI wafer. Moreover, the authors adopted a polyelectrolyte multilayer deposition method to determine the proposed sensor response to the bound mass. In this way, the maximum distance for the biomolecules to be captured and detected on the surface was determined, and the appropriate surface conjugation method could be chosen. 

Liang et al. [14] also took advantage of the antibody-antigen locking mechanism. In 2013 the authors indicated that the resonance shift would increase with a decrease in optical mode volume. Thus, a PhC nanobeam cavity with high *Q* on the order of 10^4^ and small mode volume of wavelength scale was used for further investigations. The target carcinoembryonic antigen (CEA) was a biomarker for tumors in the colon cancer treatment process. Before selectively detecting CEA, the authors first modified the nanobeam surface with captured anti-CEA. The resonance shift could be clearly observed starting from a CEA concentration of 0.1 pg/mL. However, a CEA concentration above 10 µg/mL could not induce any measurable resonance shift due to the surface saturation of physical absorption. It was remarkable that the scalable deep UV lithography on SOI wafer was adopted for fabrication. Compared with commonly used E-beam lithography process, the deep ultraviolet (UV) lithography had the advantages of high-volume production, low cost, and a fast process. It was indicated that these sample PhC chips provided a mean quality factor of 9000, which proved that scalable deep UV lithography was a reliable approach to achieve sensitive and low-cost biosensing tools. 

**Figure 12:**
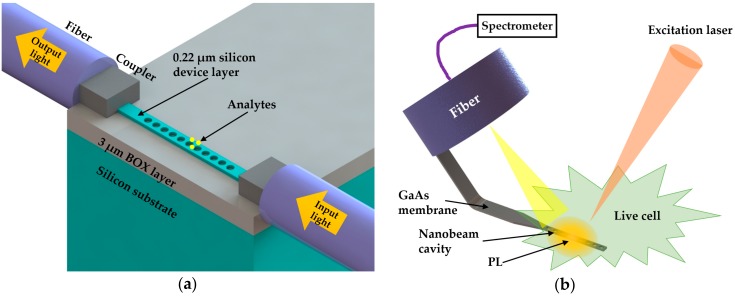
(**a**) Nanobeam cavity sensor for single streptavidin molecule; (**b**) nanobeam cavity sensor inside a live cell.

Quan et al. [15] used a single PhC nanobeam with ultra-small mode volume to investigate the detection on a single streptavidin molecule, which was only 5 nm in diameter [106]. In their experiments, the authors demonstrated that the proposed sensor was capable of detecting polystyrene particles with radii as small as 12.5 nm in DI water. To further demonstrate single molecule sensing, the authors modified the sensor surface with biotin for effective capture. They demonstrated detection of 2 pM concentration of streptavidin molecules in phosphate buffered saline (PBS) solution. Moreover, it was indicated that single molecule sensing could be improved with enhanced cavity design and laser wavelength stability in their experiments. The proposed device was fabricated on a SOI wafer with a 220 nm thick silicon device layer and a 3 µm thick BOX layer. As shown in Figure 12a, a polymer fiber-waveguide coupler was used to effectively couple the light from fiber to silicon waveguide. In this study, it was meaningful that the authors combined the perturbation theory [107] with the field distribution achieved through FDTD simulation for the theoretical prediction of the resonance shift resulted by a streptavidin molecule. 

In 2013, Shambat et al. [16] employed a PhC nanobeam cavity as a living cellular nanoprobe. The authors developed the design of nanobeam cavity based on [50]. To demonstrate the protein sensing ability, they modified the mechanical design of the probe to make it rigid enough for experiments in beakers. After chemically functionalizing the surface to achieve streptavidin-biotin binding, they demonstrated the probe capability of protein sensing. The capability of streptavidin sensing with nanoprobe could enable the possibility of further studies on label-free sensing in live cells. As shown in Figure 12b, the nanobeam cavity was fabricated on the tip of a thin GaAs membrane that was bonded to the edge of a fiber. In their experiment, a laser was used to pump the cell sample through an objective lens. As the GaAs membrane contained layers of QDs at high density, photoluminescence (PL) would be emitted, which formed a resonant mode in the cavity. PL readout was available through the fiber bonded with the nanobeam cavity. In experiments, the authors demonstrated the sound optical performance of nanobeam resonator inside the living cell. Interestingly, it was found that a cell with inserted nanobeam could survive over one week with normal cellular activities. 

Nanoparticle detection has also been reported in a gaseous environment. A gas sensor for chemical sensing based on a waveguide-coupled nanobeam cavity with chemical functionalization on the surface was presented by Chen et al. in 2014 [108]. With a fluoroalcohol polysiloxanes polymer coated on the device surface, reversible and robust binding with a target MeS molecule could be achieved. However, the excessive optical absorption of the coating caused a great reduction in Q factor at the same time. As a result, a detection limit of 1.5 ppb was demonstrated in ambient environment. 

### 4.2. Unfunctionalized Coating Surface

As introduced above, outstanding results of nanoparticle detection including enhanced selectivity have been achieved through resonator surface binding. This typically requires an additional process to capture the nanoparticles, including antibodies, chemical, and other types of functionalized surfaces. However, the shortcoming of this approach has also been recognized.

**Figure 13:**
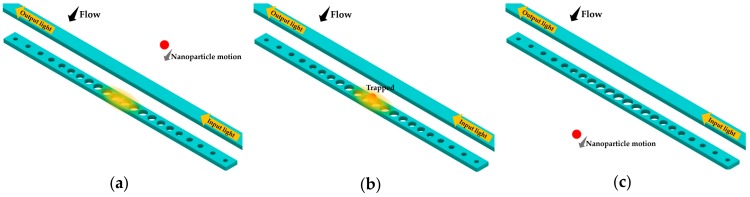
Schematics of the trap and release for nanoparticle in time sequence: (**a**) nanoparticle in flowing sample with laser power on; (**b**) nanoparticle trapped in the area where the optical field is the strongest; (**c**) nanoparticle released after laser power off.

With the unwanted optical absorption of the coating, the performance of the microcavity degrades. Moreover, the optical sensors cannot be reused due to the chemical reaction on the surface, which also increases the sensing cost. To overcome these drawbacks, research has been carried out to investigate the nanoparticle manipulation with optical trapping forces based on the PhC nanobeam cavities without functionalized coatings [17,18,19]. With an appropriate design, a PhC nanobeam cavity can confine a strong light field in a small mode volume. The intense evanescent field with a large gradient yields promising optical forces to capture nanoparticles on the resonator surface. As shown in Figure 13, the waveguide-coupled nanobeam cavity [18] is capable of capturing nanoparticles in an aqueous flowing sample.

A PhC nanobeam cavity with a central nanoslot for nanoparticle trapping was presented numerically by Lin et al. in 2009 [17]. Compared with traditional waveguide trapping devices, the optical trapping force of the proposed cavity was greatly enhanced. In 2010 Mandal et al. [18] demonstrated the optical trapping of dielectric nanoparticles as small as 62 nm and 48 nm based on a nanobeam cavity side-coupled with a waveguide. Previously, the capability of biomolecular detection based on this resonator design was demonstrated by the same group using the functionalized surface approach [13]. In their study [18], they presented the capability of trapping, storing, and rejecting nanoparticles in a microfluid channel using the optical trapping force, which enabled the possibility of an integrated biomolecule detection platform for simultaneous probing, sensing, and manipulation. However, it was also indicated that the optical trapping for a single molecule had not yet been realized due to the low *Q* factor of the nanobeam resonator resulting from the optical absorption of water.

Research efforts have therefore been made to reduce the optical absorption of water. Chen et al. [19] proposed a PhC nanobeam cavity tweezer for the manipulation of nanoparticles in 2012. The device was designed with silicon nitride, working at a resonance wavelength of 1064 nm. In this way, the optical absorption of water was greatly reduced compared with a typical design based on silicon material working at around 1550 nm wavelength. Additionally, silicon nitride has less RI contrast between the nanobeam cavity and background medium, which could extend the tail of the evanescent field. This proposed device enabled the manipulation of nanoparticles as small as Wilson disease proteins, QDs, and 22 nm polymer particles. What is more, the authors investigated the temperature effect during operation due to the optical absorption in the region of the strongest electric field. This would generate a temperature gradient and further reduce the migration of nanoparticles towards the warmest region [109]. The increase of temperature in the sample solution caused by the silicon nitride device was found to be lower than 0.3 K with the use of finite element method simulation under experimental conditions. 

Using optical trapping force, Lin et al. [110] in 2013 also demonstrated the sensing of polystyrene nanoparticles and green fluorescent protein (GFP) based on a reusable silicon waveguide-coupled nanobeam cavity. For the protein sensing, the functionalized polystyrene particles instead of functionalized resonator surface were used as carriers. These carriers were coated with antibodies to aggregate target protein molecules into clusters. Due to their large dimensions, the clusters could easily be trapped on the cavity surface through optical trapping force. These nanoparticles coated with antibodies were added to a GFP solution of different concentrations. As the probability of a nanoparticle combining with a GFP molecule was higher in the high-concentration GFP samples, there would be a large fraction of clusters and a small fraction of single particles after the carriers were added in. Since the single particle and cluster would induce different step sizes of resonance shifts, the GFP concentration could be statistically analyzed through the measurement of the percentage of single particles. The proposed device was fabricated on a SOI wafer with a silicon device layer of thickness 220 nm. Moreover, it was interesting that the authors experimentally compared the sensing performance of a waveguide-coupled micro-donut resonator and a nanobeam cavity for polystyrene nanoparticles. Even though the micro-donut resonator had a higher Q factor of 9000 compared with the nanobeam cavity *Q* factor of only 2000, the nanobeam cavity could generate a larger resonance shift and detect smaller nanoparticles having a diameter of 110 nm due to its smaller mode volume. 

In addition, research has been carried out on the detection of gold nanoparticles due to their use as biosensing labels. The sensitive detection of gold nanoparticles enables the detection of potential labeled analytes, such as DNA, proteins, and antigens. Schmidt et al. [12] demonstrated the detection of gold nanoparticles as small as 10 nm in diameter with a density of 1.25 particles per 0.04 μm^2^. A nanobeam cavity with a *Q* factor of about 180 was designed based on [35,69]. The strongly confined light field in the nanobeam cavity enhanced the effective cross section of gold nanoparticles. The proposed device was fabricated on a SOI wafer. In their experimental demonstration, a small amount of 10 nm gold particles were added to a water-based solution and subsequently deposited on the top surface of the cavity after the solution evaporated. It was indicated that there was a transmission loss due to the absorption of gold nanoparticles. 

Liang and Quan [8] demonstrated the detection of 1.8 nm diameter single gold nanoparticles in 2015. The proposed device was fabricated on a SOI wafer with 220 nm thick silicon device layer. The authors used two methods, namely piezospray and electrospray, in their experimental demonstration for single particle detection. After they are evaporated and deposited on the PhC nanobeam cavity with piezospray, the gold particles would be trapped in the center of the nanobeam cavity where the optical field was the strongest due to the optical trapping effect. In this way, a single-step resonance shift could be measured. Moreover, the authors adopted the electrospray method to demonstrate their detection capability in a flowing sample. The electrospray-generated aerosol nanoparticles had high kinetic energies that could not be easily trapped by the optical field. Thus, the detection was based on further statistical analysis of distributed resonance shifts instead of a single-step resonance shift.

Research is also ongoing for developing new sensing mechanisms aiming at single molecule detection [111,112]. For example, in 2012 Lin et al. [112] theoretically proposed a photothermal sensor that was capable of detection of a single molecule. There were two parallel nanobeam cavities in their design, with one acting as a pump and the other as a probe. The light pumped in the nanobeam cavity was tuned to the characteristic absorption line of target molecules. Thus, the temperature of the suspended nanobeam cavities would increase due to the heat generated from the absorption process. Furthermore, the resonance shift induced by the thermo-optic effect could be measured in the probe nanobeam cavity to evaluate the molecule concentration. The detection limit of gas concentration was numerically calculated to be 1.7 ppb.

### 4.3. Discussion

In general, for both the RI sensing and nanoparticle sensing applications introduced above, the transmission spectrum of the nanobeam cavity is measured to monitor the resonance shift. Most of the studies are in pursuit of nanobeam cavities with high Q factor and high sensitivity in principle. However, the sensing performance of these highly sensitive nanobeam cavity sensors may be influenced by several factors in practice [15]. Several factors, such as temperature change, chip oxidation, and solvent deposition, may cause considerable resonance fluctuations due to the highly sensitive performance. Thus, some studies have been carried out on the on-chip stabilization of cavity resonance. In [6], the authors characterized the ambient temperature effect on the fluctuations of resonance shift during measurements. Moreover, some researchers mentioned the use of on-chip thermal-stabilized reference nanobeam cavity [26,27]. Furthermore, some researchers paid attention to the sample environment effects on the degradation of sensor behavior [16,113]. In [16], the authors made use of an alumina/zirconia coating to prevent the photoinduced oxidation of device. Due to the dense layer of hydroxyl groups on the surface of silica, the silica resonators are easily to be degraded through the attraction of water in the air. To avoid degradation, the authors made use of a SiO_X_N_Y_ layer to fabricate the device [113]. In this way, the resonance and Q factor of the cavity could be stabilized. In [114], an on-chip NEMS actuator was used to stabilize the optical mode wavelength.

Normally, the resonance shift is monitored in real time for the measurement of nanoparticle sensing. An overall slope of resonance shifts versus time can be used to analyze target nanoparticles in liquid sample. However, both the analyte and solvent contribute to the slope. Since the nanoparticle binding on the cavity surface could result in resonance jump during the monitoring process, many researchers observed the fluctuations on the slope to distinguish the resonance shift caused by target nanoparticle binding on the surface (analyte) and homogenous RI change in solution (solvent) [115,116]. In this way, different types and sizes of nanoparticles could be distinguished based on the particular step size of resonance shifts in the fluctuations. In [115], the authors analyzed the resonance shift fluctuations and showed a histogram of resonance shift versus nanoparticle size. In the histogram, polystyrene nanobeads with a radius of 12.5 nm, 25 nm, or 50 nm could be inferred from resonance shift maxima. In [8,14,15], the authors also used this strategy to analyze the resonance shifts.

In summary, the ability of PhC nanobeam cavities to capture nanoparticles on cavity surfaces is essential for the further improvement of nanoparticle sensing capability. To detect flowing nanoparticles in aqueous samples, large optical gradient forces are demanded for successful optical trapping of nanoparticles. Hence, further optimization of cavity design might be needed. Moreover, there is a need for further studies on the functionalized coating of the sensor to reduce optical absorption, simplify the functionalization process, and improve sustainability.

## 5. Optomechanical Sensors

In addition to the chemical/nanoparticle sensors described in the above sections, PhC nanocavities can also be utilized for physical sensors, which rely on the interaction between optical field and nanomechanical motion [117]. The explorations of such optomechanical interactions at the nanoscale potentially enable the highly sensitive optical detection of displacement, force, and mass [118]. 

**Figure 14:**
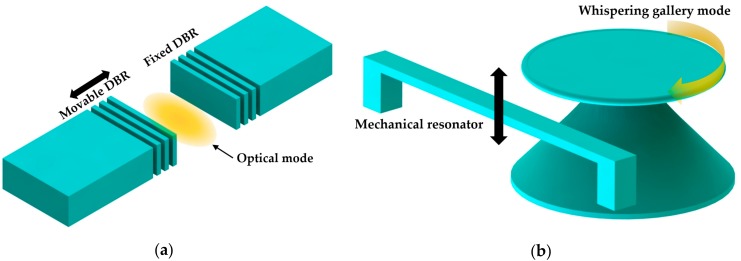
Schematics of typical optomechanical systems based on F-P cavity and WGM cavity: (**a**) an F-P cavity coupled with a mechanical resonator; (**b**) a WGM cavity coupled with a mechanical resonator.

There are several typical optomechanical systems in these studies. Various types of optical resonator are used, including F-P cavities, WGM resonators, and photonic crystal cavities. Due to their properties of simple structure and high finesse, F-P cavities are widely used at the micro scale. As an on-chip optomechanical system, two mirrors are integrated on a chip including a fixed mirror and a movable mirror. A distributed Bragg reflectors (DBRs)-based F-P cavity is one of the most typical structures [119,120]. As shown in Figure 14a, one DBR is mechanically movable and the other is fixed. However, miniaturization of such F-P cavities at the micro/nano scale generally leads to limited *Q* factor and sensitivity in sensing applications. WGM cavities represent a more viable solution for on-chip miniaturization. There are generally two ways to introduce optomechanical interactions in WGM cavities. One is to modify its own structure, such as double-layered structures [121], and the other is to design a combined structure by introducing an extra mechanical resonator such as a cantilever or a bridge beam coupled to the WGM cavity [122,123], as shown in Figure 14b. 

PhC cavity devices exhibit high optical quality factors and ultra-small mode volumes, which promise strong optomechanical effects. These optomechanical effects have been demonstrated using both 2D [124,125] and 1D [20,126] PhC cavities. Compared with 2D PhC cavities, 1D PhC nanobeam cavities offer a potentially smaller physical footprint and more flexibility in design, which make them attractive for optomechanical sensing.

In optomechanical systems, optical field and mechanical motion are coupled. Thus, the mechanical motion can be easily detected through the measurement of the light transmitted through the optical cavity. For a microcavity of optical resonance frequency (*ω_C_*) and cavity length (*x*), the optomechanical coupling coefficient can be defined as *g_om_* = *dω_C_*/*dx*, which represents the relationship between the resonance frequency shift and mechanical deformation of the cavity. Many sensing applications such as acceleration, magnetic field, etc. are based on displacement sensing because these physical variations can be converted into the displacement of a sensing component. Below, we briefly highlight the potential configurations that could be used for sensing nanoscale displacements using PhC nanobeam cavities. 

**Figure 15:**
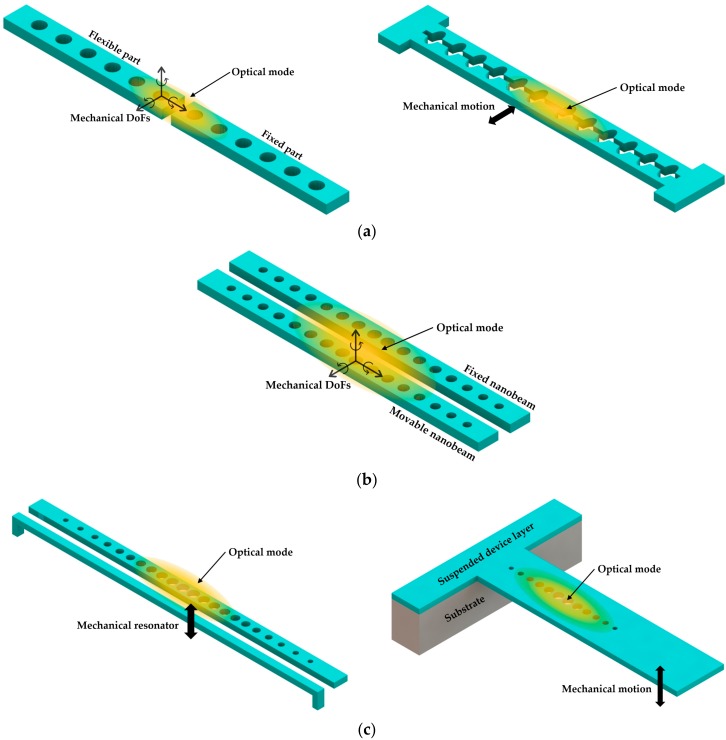
Schematics of potential optomechanical systems based on PhC nanobeam cavities: (**a**) single nanobeam cavity; (**b**) coupled nanobeam cavities; (**c**) nanobeam cavity coupled with a mechanical structure.

There are various approaches to form optomechanical sensors based on PhC nanobeam cavities, which typically fall into three categories, single nanobeam cavity, coupled nanobeam cavities, and nanobeam cavities coupled with mechanical resonators. The single nanobeam optomechanical sensors can be realized with a slice introduced in the middle or a split along the beam length direction in the center, as shown in Figure 15a. This separates the nanobeam cavity into two parts, one fixed and the other movable, and the motion of the movable part can be induced by various physical measurands. This approach creates a deformable PhC nanobeam cavity, of which resonance wavelength and Q factor can be affected by the mechanical motion of the movable part. The coupled nanobeam optomechanical sensor can be achieved through arranging two PhC nanobeam cavities in parallel formation, allowing them to be optically coupled as shown in Figure 15b. One nanobeam cavity can be fixed, while the other can be movable. The mechanical movement changes the coupling strength, thereby generating resonance shifts of the symmetric and anti-symmetric supermodes of the coupled cavities. In the third configuration, the PhC nanobeam cavity is coupled with a mechanical structure or resonator, as shown in Figure 15c. Such a mechanical resonator can be extrinsic, as shown on the left side, or intrinsic, as shown on the right side. The motion of the mechanical resonator thus modulates the PhC nanobeam cavity in terms of inducing resonance shift or *Q* factor change. With a proper design, the on-chip nanobeam cavities are able to achieve sensitive mechanical motion detection for torsion, rotation, and translation. Similar sensing mechanisms can be constructed based on other types of optical resonators [127,128,129,130,131]. To keep the paper concise, we only focus on various sensors using single nanobeam cavity and coupled nanobeam cavities below.

### 5.1. Single Nanobeam Cavity

**Figure 16:**
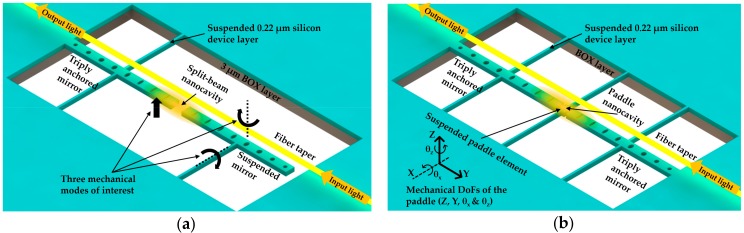
Optomechanical sensors based on split nanobeam cavity: (**a**) nanocavity torque sensor; (**b**) optomechanical paddle cavities.

Wu et al. [22] demonstrated an optomechanical torque sensor based on a PhC split-beam cavity in 2014. As shown in Figure 16a, there were two patterned nanobeams serving as optical mirrors to confine high-*Q* mode between them. The nanobeams were also suspended as cantilever mechanical resonators to support independent mechanical motion. Through engineering the holes from elliptical to circle shape [132], the authors achieved the confinement of light field in the central gap between these two nanobeams. With experiments in low vacuum and ambient conditions, the sensitivities of the optomechanical torque detection were determined to be 1.3 × 10^−21^ Nm·Hz^−1/2^ and 1.2 × 10^−20^ Nm·Hz^−1/2^, respectively. The demonstrated sensitivity was comparable with the magnetic tweezer torque sensor reported in [133]. The proposed device was fabricated on an SOI wafer with 220 nm thick silicon device layer and 3 µm thick BOX layer. A dimpled optical fiber taper was used to couple the light into the cavity. With the unique property of split beam cavities, the authors investigated not only the dispersive coupling resulting from the cavity gap modified by mechanical motions but also the dissipative optomechanical coupling [134]. The latter was strongly dependent on the photon decay rate inside the cavity gap. These interferences between dispersive and dissipative coupling were observed through measuring the transmission fluctuations. In the transmission spectrum, the optical response with dispersive coupling was resonance shift, and the optical response with dissipative coupling was a change of line width. 

Later, Kaviani et al. [23] presented strong nonlinear optomechanical coupling by modifying the abovementioned split-beam nanocavities. As illustrated in Figure 16b, with a combination of the design principles of the membrane-in-the-middle (MiM) cavities [129] and nanobeam optomechanical cavities [20], a “paddle” component was suspended inside the mirror gap between two suspended PhC nanobeam optical mirrors. In this way, the mechanical resonance of the paddle element could modify the dynamics of the optical mode confined inside the mirror gap. Due to the unique properties of optical confinement, wide free spectral range of mechanical resonance, and low mass of the whole design, the proposed optomechanical nanobeam cavity theoretically enabled the observation of thermally driven motion at a temperature around 50 mK in the device. 

**Figure 17:**
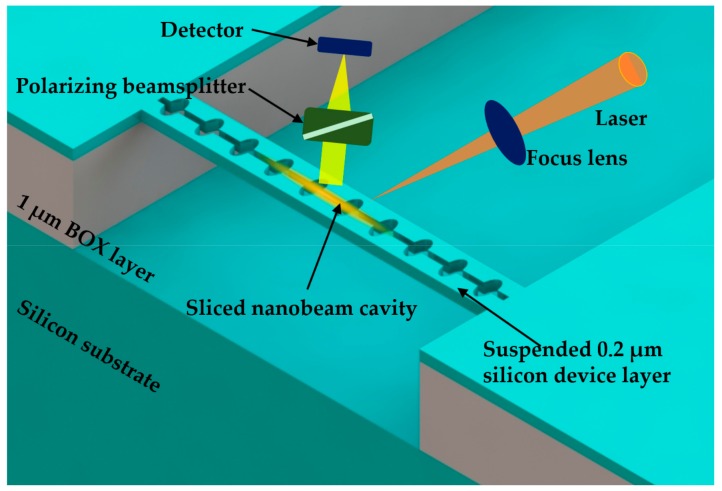
Sliced nanobeam optomechanical system with a free-space experimental setup.

Leijssen and Verhagen [24] reported a sliced PhC nanobeam optomechanical system in 2015. As shown in Figure 17, the sliced PhC nanobeam cavity was split down the middle, which formed two doubly clamped beams joined at their supports. Due to the slice being introduced as a subwavelength dielectric discontinuity, a high concentration of light energy in the subwavelength gap could be realized, which enabled a high rate of optomechanical coupling with low mass mechanical components. After analyzing the experimental results of optical radiation pressure and motion transduction, the authors reported that the photon-phonon coupling rate was as high as 11.5 MHz. The proposed device was fabricated on a SOI wafer with a silicon device layer of 200 nm thick. The free-space readout method was used for experimental detection, which provided a coupling rate comparable with the standard fiber taper coupling approach. The laser beam was focused on the cavity through an aspheric lens. To reject the directly reflecting light and detect the light coupled to the cavity, a polarizing beamsplitter was used. These investigations on large optomechanical interaction can potentially realize the detection of thermally driven displacement with noise even below the standard quantum limit [135]. 

### 5.2. Coupled Nanobeam Cavities

Eichenfield et al. [20] demonstrated the potential use of coupled PhC nanobeam cavities for optomechanical sensing. These two coupled PhC nanobeam cavities were named a “zipper cavity” [136] by the authors due to the fact that they work like a mechanical fastener, allowing sensing and actuation via the confined optical cavity field. With the use of the optical fiber taper coupler for excitation and probing, the Si_3_N_4_ zipper cavity was measured to have a high *Q* factor in the range of 10^4^ to 10^5^ in experiments. Compared with high-finesse glass microtoroid structures or F-P cavities [137], the optomechanical coupling accomplished with the zipper cavity was increased greatly. Besides the sensitive detection of mechanical displacement achieved, the mechanical motion could also be driven through the zipper cavity’s internal optical field.

**Figure 18:**
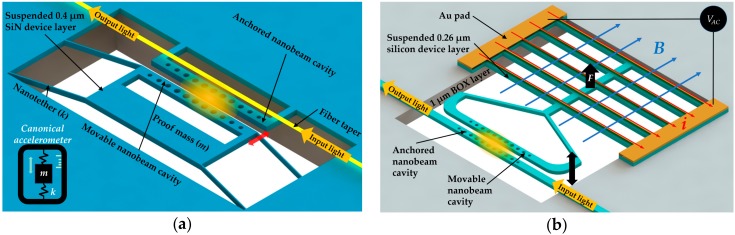
Optomechanical sensors based on coupled nanobeam cavities: (**a**) high-solution optomechanical accelerometer; (**b**) magnetic field sensor.

Using the zipper cavity as displacement readout, in 2012 Krause et al. [21] demonstrated an optomechanical accelerometer with excellent performance. The zipper cavity device realized a bandwidth over 20 kHz, an acceleration detection resolution of 10 µg·Hz^−1/2^, and a dynamic range over 40 dB, which were comparable to commercial sensors. The acceleration resolution could be quantified as noise-equivalent acceleration, which required a maximization of mass and mechanical *Q* factor product. However, there is a natural tradeoff between band width and resolution for most of the commercial accelerometers. The intrinsically low mechanical *Q* factor required a large test mass for better resolution, but a large test mass would limit the device resonance frequency and thus reduce the bandwidth. On the other hand, the zipper cavity device was capable of achieving both high resolution and wide bandwidth because the nanogram test mass with nanotether suspension brought both low mass and high mechanical *Q* factor of 10^6^. Moreover, the large optical radiation pressure force in optomechanical zipper devices enabled the control of sensor bandwidth with the optical spring effect [137]. As shown in Figure 18a, the proposed device was fabricated in a SiN layer of 400 nm thickness above a 500 µm silicon layer. The fiber taper was used to couple lightinto cavity. 

In addition, a magnetic field sensor based on similar coupled nanobeam cavities with wide operation bandwidth of 160 Hz and small footprint was demonstrated by Du et al. [25] in 2017. The authors experimentally demonstrated the sensitivity of 22.9 mV/T and resolution of 48.1 µT·Hz^−1/2^. As illustrated in Figure 18b, one of the coupled nanobeam cavities was fixed for guiding the input and output light, and the other was suspended and connected with a silicon bridge structure deposited in a thin gold layer. After being applied with an AC voltage at the structure’s mechanical resonance frequency, the gold wires as current carriers yielded a mechanical oscillation of the bridge structure in the magnetic field parallel to the device surface, as shown. This could further induce the resonance shift of a selected supermode of the coupled cavities, and subsequently affect the light intensity output. 

### 5.3. Discussion

Given the above studies, there are various sensing mechanisms based on the PhC nanobeam cavities. In general, the RI sensors introduced in previous sections are appropriate to use for biochemical sensing. These well-designed nanobeam sensors are highly sensitive to RI changes even in an aqueous environment, which makes them useful for water-based clinical samples. On the other hand, the optomechanical sensors mentioned in this section are preferred as physical sensors. Through proper design of on-chip device structure, the target physical quantity can be converted into on-chip mechanical motion. Then, the optical measurements of the optomechanical cavity can be used to detect the mechanical motion and thus quantify the target physical signal. With the use of optomechanical sensors, various physical signals, such as acceleration, magnetic field, torsion, temperature, etc., are possible to detect with ultra-high sensitivity and a low detection limit. To further develop the optomechanical sensors, it is crucial to optimize the design of the optomechanical systems to yield large optomechanical coupling coefficients. 

## 6. Temperature Sensors

Temperature sensors play a significant role in various application areas, such as automobiles [138], environmental control in buildings [139], medicine [140], and manufacturing [141]. For the past century, the resistance thermometer has been a prevailing choice for accurate measurement [142]. Though temperature uncertainties below 10 mK can be realized using resistance thermometers, they require frequent, time-consuming, and expensive calibrations due to the sensitivity to mechanical shocks. On the other hand, due to the immunity to mechanical shock and electromagnetic interference, there has been growing interest in optical temperature sensors as a substitute to resistance thermometers in recent years. Moreover, temperature sensors based on photonic structures have the advantages of a small footprint, flexible on-chip integration, complementary metal-oxide-semiconductor (CMOS) compatibility, and fast response.

Photonic temperature sensors utilize a combination of thermo-optic effect and thermal expansion. Hence, the RI and structure of PhC cavity are altered by temperature variations, which further induce resonance shifts of the cavity. Thus, the temperature variations can be evaluated from the measurement of the resonance shift. Both the thermo-optic effect *Δλ_T_* and the thermal expansion effect *Δλ_L_* contribute to the overall resonance wavelength shift *Δλ*, which is given as [143,144]:

(7) $$\Delta \lambda =\Delta \lambda_{T}+\Delta \lambda_{L}=\alpha_{W}\frac{n_{eff}}{n_{g}}\lambda \Delta T+\frac{\sigma_{T}}{n_{g}}\lambda \Delta T,\;\mathrm{where}\;\sigma_{T}=\frac{\partial n_{eff}}{\partial T}$$

where *α_w_* is the thermal expansion coefficient, *n_g_* and *n_eff_* are the group index and effective index of the photonic resonator, respectively, *ΔT* is the temperature variation, *λ* is the resonance wavelength, and *σ_T_* is the rate of effective index change with temperature.

Many photonic temperature sensors have been reported to have outstanding performance, including waveguide Bragg gratings [145], micro-ring resonators [146,147], and PhC nanobeam cavities [26,27,90,148]. Due to the small footprint and easy on-chip integration, a PhC nanobeam cavity is an outstanding candidate for temperature sensing. In 2015, Klimov et al. [149] demonstrated silicon PhC nanobeam cavity thermometers. The waveguide-coupled nanobeam cavities were cladded separately with a silicon dioxide layer and a PMMA layer, which demonstrated their corresponding sensitivity of 83 pm/°C and 68 pm/°C, respectively. The proposed device was fabricated on an SOI wafer with a 220 nm thick silicon device layer and 3 µm thick BOX layer. The one with a PMMA layer had lower sensitivity due to the negative thermo-optic coefficient of PMMA. The sensitivity of the PhC temperature sensor is mainly limited by the thermo-optic coefficient of the material used. Silicon, the most common material for photonic devices, has a thermo-optic coefficient of only 1.8 × 10^−4^ [150]. 

**Figure 19:**
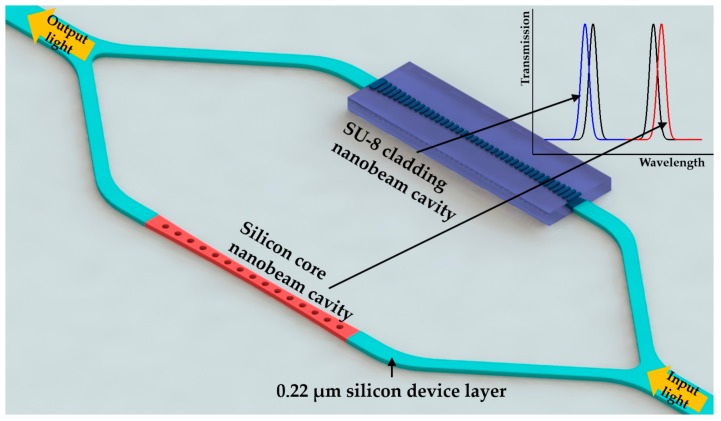
Sensitive temperature sensor based on nanobeam cavities (silicon core of positive thermo-optic coefficient in red and SU8 cladding of negative TO coefficient in blue).

Taking advantage of the Vernier effect, in 2016 Kim and Yu [147] demonstrated a temperature sensor based on cascaded ring resonators with a high sensitivity of 293.9 pm/°C, but the large envelope peak fitting error made the detection limit 0.18 °C. Zhang et al. [26] utilized the parallel nanobeam cavities [151,152] as shown in Figure 19. They demonstrated a temperature sensor with a sensitivity of 162.9 pm/°C. The detection limit was calculated to be 0.08 °C using the definition provided in [61]. To better utilize the thermo-optic property of materials, the authors designed two different types of nanobeam cavity [6,153]. One of them was cladded with SU-8 and the other had no cladding. Through a proper design, the SU-8 cladded nanobeam cavity could localize 70% of light field in the SU-8 region. This contributed to the increase of the resonance blue shift due to the negative thermo-optic coefficient of SU-8. Meanwhile, the other nanobeam cavity was designed to confine light in the silicon core, which would yield a red shift. The schematic of the device is shown in Figure 19. The sensitivities of these two nanobeam cavities were reported to be −99 pm/°C and 63.9 pm/°C, respectively. Thus, the overall sensitivity of the proposed device was increased to 162.9 pm/°C. What is more, based on the different responses to surrounding RI change and the temperature variation of the two cascaded nanobeam cavities, the simultaneous sensing of temperature and RI was demonstrated by Liu and Shi in 2017 [27]. 

The ultra-small footprint of a nanobeam cavity makes it an ideal candidate as a temperature reference sensor for on-chip integration with other types of sensors. Due to the fluctuations of ambient temperature, signals for sensors might deviate from their ideal calibrated performance. Thus, additional approaches such as the use of a thermo-electric cooler and cooling fluid [154] are often required to eliminate the deviation induced by the temperature difference, which obviously increases the cost and design complexity. On the other hand, with an on-chip integrated temperature sensor, the temperature fluctuations can be compensated for effectively during operations.

## 7. Conclusions

To be implemented in practice, these PhC nanobeam sensors pose technical challenges in manufacturing. The ability to pattern features smaller than 100 nm is required for the realization of these PhC nanobeam sensors for near-infrared wavelengths. As both of the planar PhC structures and the commercial semiconductor devices rely on planar pattern transfer, it makes the commercially available fabrication processes ideally suited for the planar PhC devices. Moreover, as the mass production of 7 nm semiconductor devices has started in 2018, the commercial processes can satisfy the dimension requirement of PhC structures. Leveraging the CMOS-compatible technology, the silicon photonics chip can be fabricated cost-effectively. However, efforts still need to be made to realize the high-yield manufacture of these photonics sensors. The stability of the PhC resonator is significantly influenced by fabrication errors. Fabrication errors in the position and size of the PhC structures may result in fluctuations of resonance wavelength and *Q* factor [155]. Thus, repeatable and stable silicon photonics processes are expected for sensor manufacturing. Moreover, as optical testing for reliability is crucial for the manufacturability, further work from foundries will be needed to develop photonics testing capability [156]. In addition, challenges remain in the packaging for these photonics sensors, such as fiber coupling, laser source, and efficient thermal management [157]. As silicon photonics has been one of the outstanding technical solutions for many applications, such as optical sensing, optical communications, and on-chip optical interconnections, there is a need for further studies and industry efforts to realize high-yield chip manufacturing.

In this paper, a comprehensive review of the PhC nanobeam cavity-based sensors is presented. In recent years, a significant amount of work on PhC nanobeam cavity sensors has been carried out, which demonstrates the promising role of PhC nanobeam cavities among various other types of optical cavities for sensing applications. Here, only the uses of PhC nanobeam cavities in RI sensing, nanoparticle sensing, optomechanical sensing, and temperature sensing are highlighted. We summarized their sensing principles, typical designs, and key developments in the recent past. Other sensing applications of the PhC nanobeam cavities also exist, and their working principles and design methods are similar to the approaches reviewed here. In the future, with technological advancements in cavity design and fabrication process, sensors based on PhC nanobeam cavities with further enhanced sensing performance can be expected. Additionally, due to their advantages (small footprint, ultra-sensitive optical readout, and ease of on-chip integration), new types of sensor-based PhC nanobeam cavities might be identified and explored. In conclusion, PhC nanobeam cavities offer a suitable platform for sensing applications and have significant potential in a range of practical areas.
